# Cell–matrix and cell–cell interaction mechanics in guiding migration

**DOI:** 10.1042/BST20230211

**Published:** 2023-08-23

**Authors:** Hoang Anh Le, Roberto Mayor

**Affiliations:** Department of Cell and Developmental Biology, University College London, Gower Street, London WC1E 6BT, U.K.

**Keywords:** cell migration, cell–cell interactions, mechanobiology, stiffness, tissue mechanics

## Abstract

Physical properties of tissue are increasingly recognised as major regulatory cues affecting cell behaviours, particularly cell migration. While these properties of the extracellular matrix have been extensively discussed, the contribution from the cellular components that make up the tissue are still poorly appreciated. In this mini-review, we will discuss two major physical components: stiffness and topology with a stronger focus on cell–cell interactions and how these can impact cell migration.

## Introduction

When cells migrate inside a multicellular body, they make extensive contact with their surrounding tissue. While biochemical signalling is important, physical forces and the mechanical properties of the tissue also contribute critical regulatory cues to the migratory behaviours of cells.

Tissues are composed of two main components: the extracellular matrix (ECM) and the cells. The ECM contains proteins, such as collagens and fibronectin, that provide structural support to the tissue. The cellular component refers to the cells that make up that particular tissue. During migration, a cell that enters into a tissue can encounter an array of physical cues, resulting in a response in its migration strategy, which we will consider in more detail below. In this mini-review, we will summarise some of the recent developments in understanding the effects of the physical environment on cell migration by taking into consideration the cell–ECM and cell–cell interactions. There have been considerable progress on the study of cell–matrix interaction, which has been reviewed elsewhere [1–4], therefore this review will focus mainly on the topic of cell–cell interactions during migration. We will draw on examples taken from a wide range of contexts in development, cancer biology and immunology to recapitulate the generality of these ideas.

## The extracellular matrix and cell migration

### The ECM stiffness

**Figure 1.**
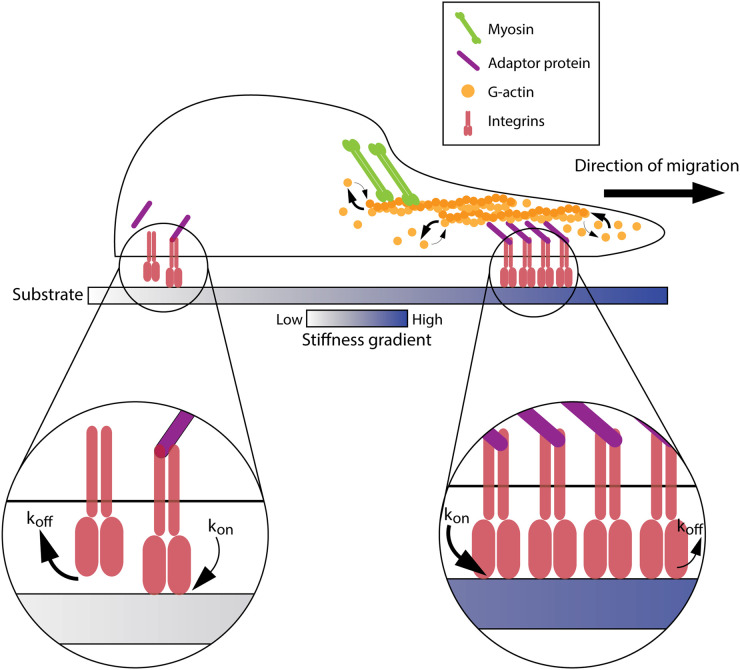
The molecular clutch model of durotaxis. Cells bias their migration towards the stiffer region due to having a higher force loading rate of integrins binding to the substrate (*k*_on_ > *k*_off_) at the front, than at their rear (*k*_off_ > *k*_on_). This allows more integrins to cluster at the leading front, hence higher actin polymerisation. Thicker arrows denote a higher rate than thinner arrows.

Much of our initial understanding of the effect of ECM stiffness on cell migration comes from using tuneable synthetic hydrogels such as polyacrylamide [5] or alginate gels [6]. By changing the concentrations of these substrates or the degree of cross-linking, the stiffness can be varied, which revealed the tendency of NIH3T3 fibroblasts [7] among other cell types [8,9], to preferentially migrate towards a stiffer substratum. This phenomenon is known as durotaxis [10] and was later explained using the molecular clutch model. In this model, there are five main components involved: the substratum, the integrins, the adaptor proteins, the filamentous actin, and the myosin motors [3,4,11–13]. The model begins with integrins binding to ECM ligands as well as connecting to the filamentous actin via adaptor proteins such as talin (Figure 1). As myosin contractility pulls on actin, this strengthens integrins’ affinity to its substrate (known as a catch-bond mechanism). The speed of this binding (also known as the force loading rate) is faster on stiffer substrates, which allows for more integrins to cluster, resulting in an increase in traction force generation, thus biasing the cells towards stiffer regions.

Recent evidence refines this model by introducing the concept of optimal stiffness [12,14,15]. It is proposed that when substrate stiffness is too high, the force loading rate occurs too rapidly, which causes an uncoordinated engagement of the different components within the molecular clutch — a phenomenon known as frictional slippage. This provides three conclusions: (1) cells may not always prefer stiffer substrates over softer ones, (2) different cell types have a specific range of optimal stiffness, and (3) cells can move downstream of a stiffness gradient to find their optimal stiffness, a phenomenon called negative durotaxis [16]. Further evidence supporting this hypothesis comes from calculating the ratio between the stiffness of the substratum and the independent stiffness of the cell. According to a new model that takes into account the ‘soft substrate effect' [17] (a phenomenon that occurs when the substrate underneath the cell being measured deforms due to the pressure of the cantilever), cells do not change their cortical stiffness based on the underlying substrate. Therefore, cells can independently compute a window of stiffness where their actin cytoskeleton machinery is able to break symmetry and become polarised for migration, even if it means going against a stiffness gradient.

Matrix stiffness has a profound effect on the migration of cancer cells. Many solid tumours are found to be stiffer than the surrounding tissue, for example, ∼150 Pa in normal *versus* 4000 Pa in breast cancer [18]. The idea of optimal stiffness could potentially explain why cancer cells leave their stiff tumour environment to invade the relatively softer normal surrounding tissue. Contrastingly, cancer cells can actively modify the stiffness of this matrix [19,20]. The network of fibres can be locally bundled up at the cell's anterior protrusions to provide traction to pull the cell forward [21]. Laser ablating just the front of this pre-strained region halts cell migration. In a stiff 3D matrix, cells have more elongated morphology compared with the more clustered phenotype observed in the soft matrix [8,20,22], which perhaps reflects the migration-inducing property of stiff matrices. In breast cancer, a stiff matrix triggers the production of the oncogenic actin-regulatory protein MENA [23], which is known for participating in invadopodia formation capable of degrading the ECM and prompting haptotaxis towards blood vessels for intravasation [24]. In pancreatic cancer, the enzyme creatine kinase B (CKB) is gradually up-regulated with stiffening substrates in a YAP-dependent manner, which is thought to provide the ATP needed for a faster actin turnover at the cell's leading front [25]. This means substrate stiffness controls how certain types of cancer can generate energy for proliferation and migration [26]. The link between mechanical cues and metabolism remains an exciting area for future exploration.

Substrate viscoelasticity is also another important factor in modulating cell migration. If a soft substrate has a fast stress relaxation rate, meaning the deformation in the ECM remains even after the applied force has disappeared (a property known as viscoelasticity), then cells can use WASP-mediated actin-rich protrusions to wedge open a path to efficiently migrate through [27]. This behaviour has been observed in monocytes [27] and fibrosarcoma cells [28] and is in parallel with observations in neutrophils [29] and dendritic cells [30] where WASP-mediated actin puncta were used to counteract matrix compression.

However, *in vitro* studies fail to recapitulate the complexity *in vivo*. Manipulating matrix stiffness *in vivo* and having the capability to verify such manipulation remain challenging tasks. Explant model systems, transparent embryos as well as the use of second harmonic generation imaging can give us a proxy for these properties in a more physiologically relevant context to bridge the gap between *in vitro* and *in vivo*.

### The matrix topology

Inside an organism, cells are often embedded in a 3D space. Hence, its topology can directly affect the cell's behaviours. One such property is porosity. Porosity refers to how much free space is available within a matrix and is defined by the ratio between the volume of the empty space over the volume of the total reservoir and is often inversely correlated with density [31].

Generally speaking, smaller pore size impedes cell migration [32]. Human foreskin fibroblasts embedded in rat tail collagen polymerised at 4°C, which coalesces into longer and thicker bundles with an overall less dense network, migrate ∼2 times faster using the pressure-based migration mode, known as lobopodia, compared with gels of the same stiffness that are polymerised at 37°C, which are less porous [33]. A similar observation was made with macrophages where these cells exhibited a slower mesenchymal morphology when embedded in a dense collagen matrix, however a switch to the faster ameboid migration in fibrillar collagen was observed [34]. A more porous matrix also means more possible paths for a cell to move through. In the case of dendritic cells, this pathfinding process proves to be a struggle for cells that have multiple filopodia, while cells with a single filopodia move the fastest and the most directional [35–37].

However, it seems that in the case of collective migration, cells can migrate more efficiently in denser matrix conditions. Cancer cells form tubular structures that mimic the vascular network in high-density collagen or low-density collagen mixed with the crowding agent polyethylene glycol (PEG) [38–40]. Tumour spheroids form more cell clusters that invade more readily into the higher-density collagen matrix. It is tempting to speculate that in the denser matrix, collective migration would be more advantageous over single-cell migration since clusters generate a higher deformation force, and thus can carve out a pathway for follower cells to move through.

**Figure 2.**
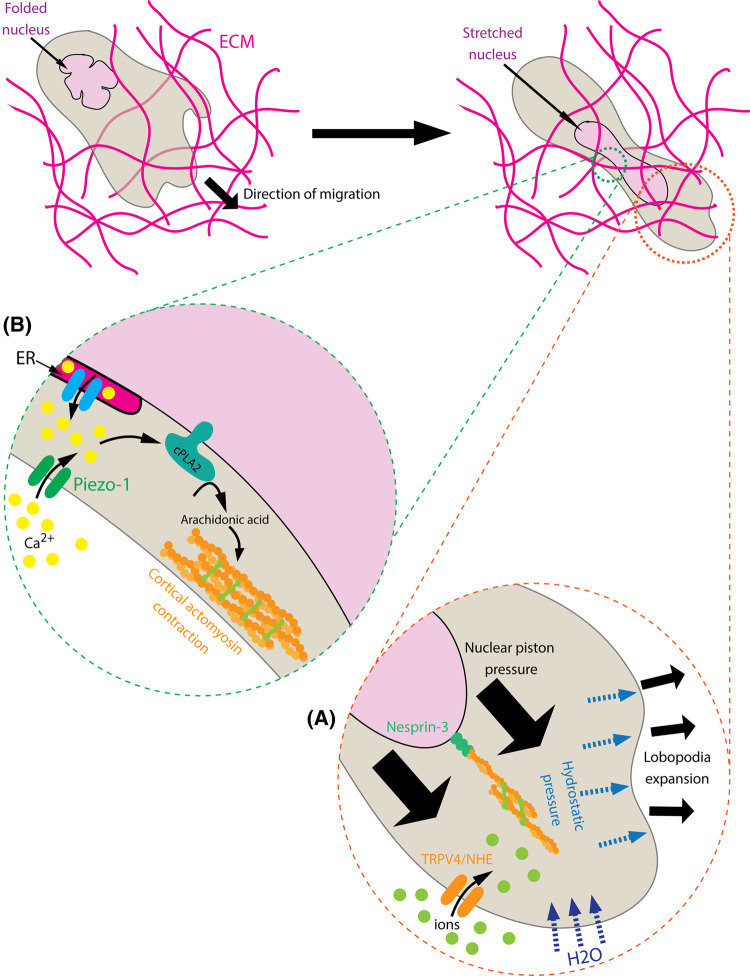
The nuclear piston and nuclear compression model allow cells to migrate through a low porous environment. Under non-constricted conditions, the nucleus has natural folds. Upon squeezing through a narrow constriction: (**A**) The nucleus is being pulled forwards by actomyosin contractility through Nesprin-3, which pressurises the front of the cell. This opens up ion channels such as TRPV4 or NHE, allows ions to flux into the cells, increases the osmotic pressure and draws in water. The influx of water causes the expansion of the front protrusion, which wedges open the matrix for the cell to pass through. (**B**) The nuclear folds are stretched, leading to the release or influx of Ca^2+^ ions into the cytoplasm through ion channels on the Endoplasmic Reticulum or on the plasma membrane. This triggers the binding of the cPLA2 enzyme to the nuclear envelope to catalyse the synthesis of Arachidonic acid, which triggers cortical actomyosin contraction and stiffens the cells to allow passing through narrow pores.

For a single cell migrating through a matrix, the most rate-limiting step is fitting the nucleus — the stiffest organelle — through the pore [41]. Multiple studies suggest that the nucleus itself is utilised as a kind of piston to aid with the migration process [42,43]. In fibroblasts, when cells are exposed to a low porosity matrix, actomyosin contractility is triggered, which pulls on the actin cytoskeleton connecting to the nucleus via Nesprin-3 towards the front [43], effectively pressurises the cytoplasm and generates lobopodial protrusions (Figure 2A). This leads to an influx of ions through opening channels such as TRPV4 and NHE-1, which increases the osmotic pressure and draws in water at the cell-front. This expands the protrusion to widen the viscoelastic matrix and allows the cell to pass through [44]. The increased contractility during constricted migration is due to the complete unfolding of the nuclear envelope, which triggers Ca^2+^ ions release from the endoplasmic reticulum or through Piezo1-mediated Ca^2+^ influx. This triggers the binding of calcium-dependent phospholipase 2 (cPLA2) to the stretched outer nuclear envelope, catalysing the synthesis of arachidonic acid which activates actomyosin contraction [45,46]. This stiffens the cell cortex and thus resists the compression from the dense matrix around it (Figure 2B). Calcium ions also suppress the activity of protein kinase A, which is a known activator of Rac1 [47]. This potentiates the elevation of Rho-ROCK signalling [48] and allows cells to enter contraction-based instead of lamellipodia-based migration. This mode of migration can be utilised for as long as the confinement site is not smaller than 10% of the nucleus's cross-section diameter before matrix protease-dependent migration is activated [41]. What controls nuclear folding is an intriguing question that has not been explored but could potentially be used to control the threshold level for how sensitive cells are to compression. It is also important to note that the mechanisms described above have only been observed *in vitro*, while *in vivo* observations are still scarce [49] and thus more research is needed.

## The cellular environment in guiding cell migration

Despite the intimate connection the cellular environment poses to cell migration, this aspect of tissue mechanics remains poorly discussed. Studying the effects of cellular mechanics on neighbouring cell migration is somewhat less common because it often requires a native tissue or an *in vivo* system. Gaining access to the tissue of interest is not always possible, and even then, imaging these interactions as well as measuring the mechanical properties of the native tissue can often be technically challenging. Despite these difficulties, recent evidence from *Xenopus*, *Drosophila* and zebrafish embryos has suggested such effects of cellular mechanics exist and can have major impacts on the migratory behaviour of neighbouring cells. In this section, we will discuss the role of cellular stiffness and tissue architecture on cell migration.

### Cellular stiffness

**Figure 3.**
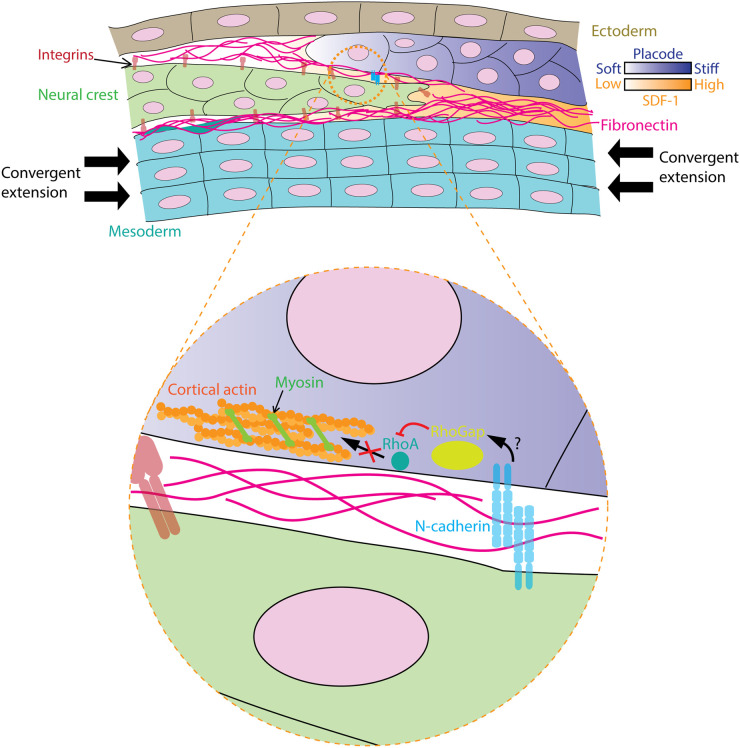
The mechanism of durotaxis *in vivo* by neural crest. Convergent extension causes mesodermal cells to pack together, increasing their cell density and therefore the tissue stiffness. This initiates the migration of neural crest cells. Neural crest also follows a chemotactic gradient of SDF-1 secreted by the placode. When the leading neural crest cells make contact with the placode through homotypic N-cadherin interactions, this potentially recruits RhoGAPs, which deactivates RhoA, and therefore lowers down actomyosin contractility, causes the placode to soften at the point of contact. This creates a stiffness gradient of the placode in the same direction as SDF-1, guiding the neural crest migration forwards.

One of the most recent pieces of evidence of *in vivo* tissue stiffness sensing comes from the study of neural crest migration in *Xenopus* embryos. Neural crest is a population of embryonic stem cells that delaminates from the neural fold and migrates along the dorsoventral axis. This migratory behaviour has been likened to cancer cell invasion during metastasis. During this process, the cell–cell adhesion molecule, E-cadherin, is down-regulated, while N-cadherin is up-regulated. Neural crest cells also follow the gradient of the chemotactic molecule SDF-1 (CXCL12) secreted by the neural placode in a chase-and-run mechanism [50], while avoiding areas with inhibitory signals such as Versican [51] or Semaphorin 3A [52]. Inside an embryo, neural crest cells migrate between two thin layers of fibronectin present on the surface of the mesoderm and placodal ectoderm (Figure 3). Intriguingly, atomic force microscopy (AFM) measurement of this mesoderm shows a progressive increase in the apparent stiffness from stage 13 to stage 20 embryos that correlates with the initiation of neural crest migration [53]. When the mesodermal layer is artificially softened by overexpressing constitutively active myosin phosphatase or by releasing the tissue tension through tissue ablation, this prevents the collective migration of the neural crest. In contrast, when the mesoderm is stiffened up via the overexpression of the constitutively active myosin light chain or by enhancing the tissue tension by pressing with the AFM cantilever, this promotes the migration of the neural crest cells. Importantly, removing the fibronectin layer has no effect on the measured stiffness, which suggests that the ECM does not have a significant mechanical contribution in this case, apart from providing an adhesive substratum. Interestingly, in contrast with the global increase in mesodermal stiffness over time, the stiffness of the placodes is not homogeneously distributed. Careful measurements of the placode reveal a dorsoventral gradient of stiffness in the same direction as the SDF-1 gradient [54]. It was proposed that the portion of the placode that is in contact with the neural crest is softened through N-cadherin signalling by reducing cortical actin. Although a detailed mechanism was not extensively discussed, it is not unreasonable to speculate that it follows previously described signalling pathways. For example, homotypic N-cadherin interaction recruits and activates RhoGTPase activating proteins (RhoGAP) like p190 [55] or Gap21/23 [56] that inhibit RhoA. This results in the reduction in cortical contractility, therefore, reduces the apparent stiffness of the placodal cells at the interface with the neural crest. It is interesting to note that convergent extension which leads to the increase in mesodermal cell packing is the driving force behind mesodermal stiffening. While the neural crest makes contact with both the placode and the mesoderm, the mechanism by which the placode expresses the stiffness gradient remains an interesting question for future studies.

In another model of retinal ganglion cells, the neurons that are part of the optic system were shown to preferentially bend towards softer tissue regions where fewer cells are packed together [57]. It was shown that the increased in tissue stiffness is due to an increase in cell density, showing again that neurons are sensing the mechanical property of the surrounding tissue and not from the ECM [58]. *In vitro*, it was shown that axons are Piezo1-dependent mechanosensitive [57,58] and are longer on stiff polyacrylamide gels compared with softer gels where they assume a more explorative morphology. This exploration behaviour was argued to be important for the axon bundle to find the optic tectum *in vivo*. However, it is unclear whether this seemingly negative durotaxis is an active or a passive process. If this was a passive process, a possible explanation would be that one side of the bundle grows faster and is more migratory compared with the other side due to the exposure to a stiffer substrate, then the bundle would naturally curve towards the softer region. If the bending was an active process, then we would expect to observe more active growth cones formed on the softer side. However, testing these hypotheses would require a higher-resolution imaging modality, which could be technically difficult *in vivo*. Nevertheless, tissue mechanical properties by cellular components play an important regulatory role in cell migration.

### Tissue architecture and topology

Similar to the ECM, the architecture of tissue can have a profound impact on cell migration. *In vitro*, synthetic substrates with altered topology have been extensively used. Recent studies show that on a wavy substrate, cells naturally fall into the troughs or negative curvatures through a mechanism termed curvotaxis [59,60]. Positive curvatures or convex structures were shown to bend and compress the nucleus, likely in coordination with stress fibres that straddle on top. Through a mechanism that is likely to be similar to what we described in the previous section [45,46], this nuclear compression triggers myosin contractility, which pushes the nucleus down into the trough.

When these curvatures are small enough, cells can use them to migrate even in the absence of any focal adhesions. When talin-knockout T-cells are placed in a smooth microchannel, they fail to migrate. However, when these same cells are placed in a serrated channel that contains repeated wavy patterns, they become mobile, albeit still slower than wild-type cells [61]. The bigger the serration, the less effective the microchannel becomes in rescuing the migration ability of the talin-knockout cells, suggesting that cells have a certain degree of resolution for substrate topology that they can feel. This mode of repetitive texture-dependent is sometimes referred to as rachetaxis [62] and seems to be the most relevant to cells that use bleb-based migration. The current proposal is that actin flowing from the front to the rear of a cell encounters resistance from the serration, which generates a countering force propelling the cell forwards. One point to note is that the tested serrated patterns were symmetrical on both sides and high in stiffness, while native tissues are often heterogenous and a lot softer. Hence, it remains to be seen whether rachetaxis is relevant *in vivo*.

A related mode of migration that is potentially more physiologically probable is frictiotaxis. As the name suggests, this migration mode relies on non-specific friction interactions between the migrating cell and its surrounding. Non-adherent Walker cells that do not naturally form focal adhesions and cannot migrate effectively on a flat 2D substrate migrate efficiently in microchannels coated with bovine serum albumin to have increased friction [63]. The same cells fail to migrate when this coating is replaced with Pluronic F127, which is known for reducing friction. Interestingly, a recent preprint suggests that by simply increasing the friction gradient, cells can be directed towards a higher friction region [64]. The proposed mechanism is that the retrograde flow of actin is resisted by the architectural interactions between random transmembrane proteins and the minuscule irregularity on the substrate wall combined with rear-end myosin contractility, which creates a propelling force driving cells forward. It is somewhat analogous to rachetaxis but instead of the cell-scale architectural topology, friction can happen at the molecular scale. It remains to be seen whether frictiotaxis occurs *in vivo*.

**Figure 4.**
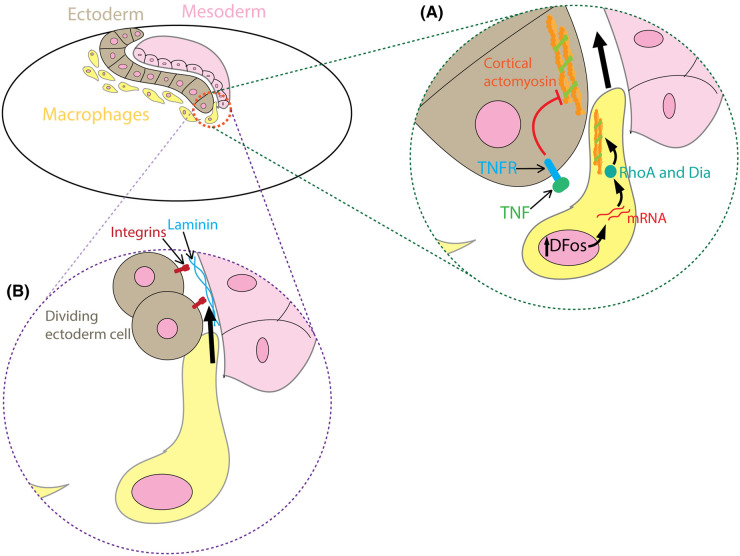
Guiding macrophage migration *in vivo* by tissue mechanics. Macrophages invade the germ band through an opening between the layers of the ectoderm and the mesoderm. (**A**) TNF secreted by the surrounding cells binds to the TNF receptor (TNFR) on the ectodermal cell. This leads to the dephosphorylation of myosin, therefore lowers down cortical actomyosin contraction and softening the cell. As the first macrophage is squeezed between the ectoderm and the mesoderm, it up-regulates the transcription factor cFos, which up-regulates mRNA of actin cross-linking proteins, which then activates RhoA and Dia to increase cortical actin polymerisation and contraction. This stiffens up the macrophage and allows it to squeeze in between the two layers of tissue. (**B**) The ectodermal cell at the entry point adheres to the Laminin covering on top of the mesoderm layer through integrins. When this cell enters cell division, it rounds up and temporarily detaches from the Laminin. This creates an opening for the macrophage to wedge in.

In *Drosophila* embryos, ectodermal tissue architecture affects macrophage invasion into the germband. The ectodermal cell that blocks the entrant gate to the germband needs to be physically moved away to let the first macrophage through (Figure 4A). Tissue necrosis factor (TNF) Eiger secreted by the surrounding cells triggers a dephosphorylation of the myosin light chain in the ectodermal cell, resulting in the loss of cortical tension and loosening of the blockage, which allows the macrophages to squeeze through [65]. The macrophages also respond to being squeezed by up-regulating the transcription factor Dfos, which leads to Rho1 and the formin Dia activation [66]. This leads to a global increase in cortical actin polymerisation within the body of the macrophage, possibly as a protection mechanism. In addition, upon rounding up during cell division, the entrance-blocking ectodermal cell temporarily loses its integrin adhesions with the laminin layer covering the mesoderm (Figure 4B), thus forms a physical opening for the macrophages to wedge in [67]. Interestingly, this division does not seem to be triggered by the macrophages, therefore the factors underlying the timing of this crucial division remains to be explored. The studies discussed in this section highlight the extensive physical regulatory mechanisms that organisms employ to control cell migration. In the same model organism, within the egg chamber, a cluster containing 2 polar cells surrounded by a few border cells migrates through a densely packed tissue of nurse cells [68,69]. When the cell–cell adhesion molecule E-cadherin is knocked down in border cells or nurse cells, this significantly reduces the directionality of the cell cluster. In contrast, when E-cadherin is overexpressed in nurse cells, this slows down the migration of the cell cluster but does not affect its directionality [70]. Through fluorescence resonance energy transfer (FRET) microscopy, it was revealed that the front of the cell cluster is constantly under tension. The homotypic E-cadherin interactions activate Rac1 at a few front cells, promotes protrusion formation, and thus forms a positive feedback loop driving directional migration of the cell cluster. Recent data also point to the role of the nucleus in the leader cell to act as a wedge to assist the migration [71], analogous to the nuclear piston model described previously.

During the early development of zebrafish embryos, the prechordal plate migrates to the animal pole, while the outer neurectoderm migrates in the opposite direction towards the vegetal pole [72]. Cell tracking reveals a characteristic vortex-liked movement of the neurectoderm in normal embryos, while the mutants that lack the endogenous mesoderm or have defective mesoderm movement, this vortex pattern is lost. It was then revealed that the physical interactions of E-cadherin between the prechordal plate and the neurectoderm are the source that gives rise to this pattern. Shearing beads coated with E-cadherin on top of a layer of ectoderm explant reproduces this vortex pattern. This phenomenon was argued to be due to the friction between the two tissue layers. However, it is interesting to note that friction-based migration occurs due to non-specific interactions of transmembrane proteins. The fact that only specifically depleting E-cadherin or the use of E-cadherin coated beads had an effect argues against the friction-based hypothesis but rather a more specific cell–cell interaction must be required. An interesting question is whether the overexpression of a random transmembrane protein could also rescue the vortex pattern. Another example for cell–cell interactions comes from the zebrafish posterior lateral line primordium as 3D imaging identifies a cell subpopulation that lies on top of the cluster that makes extensive contact with the overlying skin tissue [73]. These so-called superficial cells extend lamellipodial-liked protrusions and seemingly use the basal side of the skin as a substrate to assist with the migration of the entire lateral line cluster. Importantly, removing the skin completely abrogated this migration while an increase in the cluster's height was observed. This suggests that the skin tissue itself provides a type of compression along with being a substrate for the lateral line. *In vitro* compression has been repeatedly shown to induce blebs in different cell types [74], so whether the skin compression seen in the case actively induces the observed protrusions remains to be elucidated (Table 1).

**Table 1:** Different modes of tissue architecture and topology on cell migration

Mode	Model system	Effects
Curvotaxis	Cell lines (Mesenchymal stem cells, fibroblasts, MDCK)	Cells prefer concave over convex surface
Rachetaxis	T-cells	Cellular-scale serration rescues T-cell migration
Frictiotaxis	Walker cells	Molecular-scale non-specific friction drives forward migration Cells move towards higher friction area
Cell–cell interaction	*Drosophila melanogaster* embryos	Ectodermal cells create physical obstructions for macrophage migration Polar-border cell cluster uses E-cadherin interactions with nurse cells to drive forward migration
	Zebrafish embryos	Prechordal plate interacts with the neuroectoderm layer in opposite direction through E-cadherins
	Zebrafish posterior lateral line primordium	Superficial cells of the posterior lateral line primordium use lamellipodia to interact with the overlying skin tissue to drive cluster migration

## Outlooks

Studies of biological systems have been heavily focused on its biochemical aspect since its conception. But recent developments in the field have argued for the significant role of physics in dictating many biological phenomena. By drawing from widely different contexts and model systems, we hope we have demonstrated the importance of mechanical properties and physical interactions between cells and their tissue environment. This knowledge not only helps us gain a better understanding of how cell migration is regulated but also how we can expand our toolbox in devising strategies for when things go wrong.

We also hope that we have brought more attention to the rather lesser-discussed cellular mechanics as one of the important influencing factors. Unlike the ECM, cells are alive and are responsive to stimulations. They are active matters. Hence, any external mechanical impulses can be met with an adaptive response. This arguably can have a more diverse and complex outcome that we hope future research will be able to address.

Apart from stiffness and architecture, there are also many other physical factors that we have not discussed in this review, such as hydrostatic pressure [75–77], tissue jamming and unjamming [78], and matrix alignment [79] to name a few. While it is useful to understand each factor independently, it is essential to recognise that the observed migration behaviour of a cell in tissue may likely be a result of a combination of different properties, and that cells may use the same mechanism to adapt to different physical stimuli. Future studies should try to address how each factor influences each other and how much each of them influences a cell, particularly in an *in vivo* context.

## Perspectives

- Understanding how physical factors regulate cell migration opens doors to better understanding of many biological phenomena and therapeutic implications when these processes go wrong.
- Much of the current work in mechanobiology has been focusing on the physical properties of the ECM, while physical interactions between migrating cells and their surrounding tissue are lesser explored.
- To gain a complete understanding of how tissue regulates cell migration, future studies should intercalate the properties of both the ECM and cellular components.

## Open Access

Open access for this article was enabled by the participation of University College London in an all-inclusive *Read & Publish* agreement with Portland Press and the Biochemical Society under a transformative agreement with JISC.

## Abbreviations

AFM: atomic force microscopy
ECM: extracellular matrix
TNF: tissue necrosis factor

## References

[1] Yamada, K.M., Doyle, A.D. and Lu, J. (2022) Cell-3D matrix interactions: recent advances and opportunities. Trends Cell Biol. 32, 883–895 10.1016/j.tcb.2022.03.002 35410820 PMC9464680

[2] Yamada, K.M. and Sixt, M. (2019) Mechanisms of 3D cell migration. Nat. Rev. Mol. Cell Biol. 20, 738–752 10.1038/s41580-019-0172-9 31582855

[3] Kanchanawong, P. and Calderwood, D.A. (2023) Organization, dynamics and mechanoregulation of integrin-mediated cell–ECM adhesions. Nat. Rev. Mol. Cell Biol. 24, 142–161 10.1038/s41580-022-00531-5 36168065 PMC9892292

[4] SenGupta, S., Parent, C.A. and Bear, J.E. (2021) The principles of directed cell migration. Nat. Rev. Mol. Cell Biol. 22, 529–547 10.1038/s41580-021-00366-6 33990789 PMC8663916

[5] Denisin, A.K. and Pruitt, B.L. (2016) Tuning the range of polyacrylamide gel stiffness for mechanobiology applications. ACS Appl. Mater. Interfaces 8, 21893–21902 10.1021/acsami.5b09344 26816386

[6] Vining, K.H. and Mooney, D.J. (2017) Mechanical forces direct stem cell behaviour in development and regeneration. Nat. Rev. Mol. Cell Biol. 18, 728–742 10.1038/nrm.2017.108 29115301 PMC5803560

[7] Lo, C.M., Wang, H.B., Dembo, M. and Wang, Y.L. (2000) Cell movement is guided by the rigidity of the substrate. Biophys. J. 79, 144–152 10.1016/S0006-3495(00)76279-5 10866943 PMC1300921

[8] Acerbi, I., Cassereau, L., Dean, I., Shi, Q., Au, A., Park, C. et al. (2015) Human breast cancer invasion and aggression correlates with ECM stiffening and immune cell infiltration. Integr. Biol. (Camb) 7, 1120–1134 10.1039/c5ib00040h 25959051 PMC4593730

[9] Nicolas-Boluda, A., Vaquero, J., Vimeux, L., Guilbert, T., Barrin, S., Kantari-Mimoun, C. et al. (2021) Tumor stiffening reversion through collagen crosslinking inhibition improves T cell migration and anti-PD-1 treatment. eLife 10, e58688 10.7554/eLife.58688 34106045 PMC8203293

[10] Shellard, A. and Mayor, R. (2021) Durotaxis: the hard path from *in vitro* to *in vivo*. Dev. Cell 56, 227–239 10.1016/j.devcel.2020.11.019 33290722

[11] Ayad, N.M.E., Kaushik, S. and Weaver, V.M. (2019) Tissue mechanics, an important regulator of development and disease. Philos. Trans. R. Soc. B Biol. Sci. 374, 20180215 10.1098/rstb.2018.0215 PMC6627022 31431174

[12] Elosegui-Artola, A., Trepat, X. and Roca-Cusachs, P. (2018) Control of mechanotransduction by molecular clutch dynamics. Trends Cell Biol. 28, 356–367 10.1016/j.tcb.2018.01.008 29496292

[13] Bodor, D.L., Pönisch, W., Endres, R.G. and Paluch, E.K. (2020) Of cell shapes and motion: the physical basis of animal cell migration. Dev. Cell 52, 550–562 10.1016/j.devcel.2020.02.013 32155438

[14] Bangasser, B.L., Shamsan, G.A., Chan, C.E., Opoku, K.N., Tüzel, E., Schlichtmann, B.W. et al. (2017) Shifting the optimal stiffness for cell migration. Nat. Commun. 8, 15313 10.1038/ncomms15313 28530245 PMC5458120

[15] Marhuenda, E., Fabre, C., Zhang, C., Martin-Fernandez, M., Iskratsch, T., Saleh, A. et al. (2021) Glioma stem cells invasive phenotype at optimal stiffness is driven by MGAT5 dependent mechanosensing. J. Exp. Clin. Cancer Res. 40, 139 10.1186/s13046-021-01925-7 33894774 PMC8067292

[16] Isomursu, A., Park, K.-Y., Hou, J., Cheng, B., Mathieu, M., Shamsan, G.A. et al. (2022) Directed cell migration towards softer environments. Nat. Mater. 21, 1081–1090 10.1038/s41563-022-01294-2 35817964 PMC10712428

[17] Rheinlaender, J., Dimitracopoulos, A., Wallmeyer, B., Kronenberg, N.M., Chalut, K.J., Gather, M.C. et al. (2020) Cortical cell stiffness is independent of substrate mechanics. Nat. Mater. 19, 1019–1025 10.1038/s41563-020-0684-x 32451510 PMC7610513

[18] Paszek, M.J., Zahir, N., Johnson, K.R., Lakins, J.N., Rozenberg, G.I., Gefen, A. et al. (2005) Tensional homeostasis and the malignant phenotype. Cancer Cell 8, 241–254 10.1016/j.ccr.2005.08.010 16169468

[19] Winkler, J., Abisoye-Ogunniyan, A., Metcalf, K.J. and Werb, Z. (2020) Concepts of extracellular matrix remodelling in tumour progression and metastasis. Nat. Commun. 11, 5120 10.1038/s41467-020-18794-x 33037194 PMC7547708

[20] Girigoswami, K., Saini, D. and Girigoswami, A. (2021) Extracellular matrix remodeling and development of cancer. Stem Cell Rev. Rep. 17, 739–747 10.1007/s12015-020-10070-1 33128168

[21] Doyle, A.D., Sykora, D.J., Pacheco, G.G., Kutys, M.L. and Yamada, K.M. (2021) 3D mesenchymal cell migration is driven by anterior cellular contraction that generates an extracellular matrix prestrain. Dev. Cell 56, 826–841.e4 10.1016/j.devcel.2021.02.017 33705692 PMC8082573

[22] Wullkopf, L., West, A.-K.V., Leijnse, N., Cox, T.R., Madsen, C.D., Oddershede, L.B. et al. (2018) Cancer cells’ ability to mechanically adjust to extracellular matrix stiffness correlates with their invasive potential. Mol. Biol. Cell 29, 2378–2385 10.1091/mbc.E18-05-0319 30091653 PMC6233061

[23] Wang, W., Taufalele, P.V., Millet, M., Homsy, K., Smart, K., Berestesky, E.D. et al. (2023) Matrix stiffness regulates tumor cell intravasation through expression and ESRP1-mediated alternative splicing of MENA. Cell Rep. 42, 112338 10.1016/j.celrep.2023.112338 37027295 PMC10551051

[24] Oudin, M.J., Jonas, O., Kosciuk, T., Broye, L.C., Guido, B.C., Wyckoff, J. et al. (2016) Tumor cell-driven extracellular matrix remodeling drives haptotaxis during metastatic progression. Cancer Discov. 6, 516–531 10.1158/2159-8290.CD-15-1183 26811325 PMC4854754

[25] Papalazarou, V., Zhang, T., Paul, N.R., Juin, A., Cantini, M., Maddocks, O.D.K. et al. (2020) The creatine-phosphagen system is mechanoresponsive in pancreatic adenocarcinoma and fuels invasion and metastasis. Nat. Metab. 2, 62–80 10.1038/s42255-019-0159-z 32694686 PMC7617069

[26] Park, J.S., Burckhardt, C.J., Lazcano, R., Solis, L.M., Isogai, T., Li, L. et al. (2020) Mechanical regulation of glycolysis via cytoskeleton architecture. Nature 578, 621–626 10.1038/s41586-020-1998-1 32051585 PMC7210009

[27] Adebowale, K., Ha, B., Saraswathibhatla, A., Indana, D., Popescu, M., Demirdjian, S. et al. (2023) Monocytes use protrusive forces to generate migration paths in viscoelastic collagen-based extracellular matrices. bioRxiv 10.1101/2023.06.09.544394 PMC12207522 40523187

[28] Adebowale, K., Gong, Z., Hou, J.C., Wisdom, K.M., Garbett, D., Lee, H. et al. (2021) Enhanced substrate stress relaxation promotes filopodia-mediated cell migration. Nat. Mater. 20, 1290–1299 10.1038/s41563-021-00981-w 33875851 PMC8390443

[29] Brunetti, R.M., Kockelkoren, G., Raghavan, P., Bell, G.R.R., Britain, D., Puri, N. et al. (2022) WASP integrates substrate topology and cell polarity to guide neutrophil migration. J. Cell Biol. 221, e202104046 10.1083/jcb.202104046 34964841 PMC8719638

[30] Gaertner, F., Reis-Rodrigues, P., de Vries, I., Hons, M., Aguilera, J., Riedl, M. et al. (2022) WASp triggers mechanosensitive actin patches to facilitate immune cell migration in dense tissues. Dev. Cell 57, 47–62.e9 10.1016/j.devcel.2021.11.024 34919802 PMC8751638

[31] Matrix Porosity - an overview | ScienceDirect Topics https://www.sciencedirect.com/topics/engineering/matrix-porosity

[32] Yang, Y., Motte, S. and Kaufman, L.J. (2010) Pore size variable type I collagen gels and their interaction with glioma cells. Biomaterials 31, 5678–5688 10.1016/j.biomaterials.2010.03.039 20430434

[33] Doyle, A.D., Carvajal, N., Jin, A., Matsumoto, K. and Yamada, K.M. (2015) Local 3D matrix microenvironment regulates cell migration through spatiotemporal dynamics of contractility-dependent adhesions. Nat. Commun. 6, 8720 10.1038/ncomms9720 26548801 PMC4643399

[34] Van Goethem, E., Poincloux, R., Gauffre, F., Maridonneau-Parini, I. and Le Cabec, V. (2010) Matrix architecture dictates three-dimensional migration modes of human macrophages: differential involvement of proteases and podosome-like structures. J. Immunol. 184, 1049–1061 10.4049/jimmunol.0902223 20018633

[35] Jain, N. and Vogel, V. (2018) Spatial confinement downsizes the inflammatory response of macrophages. Nat. Mater. 17, 1134–1144 10.1038/s41563-018-0190-6 30349032 PMC6615903

[36] Wang, X., Hossain, M., Bogoslowski, A., Kubes, P. and Irimia, D. (2020) Chemotaxing neutrophils enter alternate branches at capillary bifurcations. Nat. Commun. 11, 2385 10.1038/s41467-020-15476-6 32404937 PMC7220926

[37] Leithner, A., Eichner, A., Müller, J., Reversat, A., Brown, M., Schwarz, J. et al. (2016) Diversified actin protrusions promote environmental exploration but are dispensable for locomotion of leukocytes. Nat. Cell Biol. 18, 1253–1259 10.1038/ncb3426 27775702

[38] Velez, D.O., Tsui, B., Goshia, T., Chute, C.L., Han, A., Carter, H. et al. (2017) 3D collagen architecture induces a conserved migratory and transcriptional response linked to vasculogenic mimicry. Nat. Commun. 8, 1651 10.1038/s41467-017-01556-7 29162797 PMC5698427

[39] Ilina, O., Gritsenko, P.G., Syga, S., Lippoldt, J., La Porta, C.A.M., Chepizhko, O. et al. (2020) Cell-cell adhesion and 3D matrix confinement determine jamming transitions in breast cancer invasion. Nat. Cell Biol. 22, 1103–1115 10.1038/s41556-020-0552-6 32839548 PMC7502685

[40] Plou, J., Juste-Lanas, Y., Olivares, V., del Amo, C., Borau, C. and García-Aznar, J.M. (2018) From individual to collective 3D cancer dissemination: roles of collagen concentration and TGF-β. Sci. Rep. 8, 12723 10.1038/s41598-018-30683-4 30143683 PMC6109049

[41] Wolf, K., te Lindert, M., Krause, M., Alexander, S., te Riet, J., Willis, A.L. et al. (2013) Physical limits of cell migration: control by ECM space and nuclear deformation and tuning by proteolysis and traction force. J. Cell Biol. 201, 1069–1084 10.1083/jcb.201210152 23798731 PMC3691458

[42] Petrie, R.J., Harlin, H.M., Korsak, L.I.T. and Yamada, K.M. (2016) Activating the nuclear piston mechanism of 3D migration in tumor cells. J. Cell Biol. 216, 93–100 10.1083/jcb.201605097 27998990 PMC5223602

[43] Petrie, R.J., Koo, H. and Yamada, K.M. (2014) Generation of compartmentalized pressure by a nuclear piston governs cell motility in a 3D matrix. Science 345, 1062–1065 10.1126/science.1256965 25170155 PMC5248932

[44] Lee, H.-P., Alisafaei, F., Adebawale, K., Chang, J., Shenoy, V.B. and Chaudhuri, O. (2021) The nuclear piston activates mechanosensitive ion channels to generate cell migration paths in confining microenvironments. Sci. Adv. 7, eabd4058 10.1126/sciadv.abd4058 33523987 PMC7793582

[45] Lomakin, A.J., Cattin, C.J., Cuvelier, D., Alraies, Z., Molina, M., Nader, G.P.F. et al. (2020) The nucleus acts as a ruler tailoring cell responses to spatial constraints. Science 370, eaba2894 10.1126/science.aba2894 33060332 PMC8059074

[46] Venturini, V., Pezzano, F., Català Castro, F., Häkkinen, H.-M., Jiménez-Delgado, S., Colomer-Rosell, M. et al. (2020) The nucleus measures shape changes for cellular proprioception to control dynamic cell behavior. Science 370, eaba2644 10.1126/science.aba2644 33060331

[47] Hung, W.-C., Yang, J.R., Yankaskas, C.L., Wong, B.S., Wu, P.-H., Pardo-Pastor, C. et al. (2016) Confinement sensing and signal optimization via piezo1/PKA and myosin II pathways. Cell Rep. 15, 1430–1441 10.1016/j.celrep.2016.04.035 27160899 PMC5341576

[48] Parri, M. and Chiarugi, P. (2010) Rac and Rho GTPases in cancer cell motility control. Cell Commun. Signal. 8, 23 10.1186/1478-811X-8-23 20822528 PMC2941746

[49] Soans, K.G., Ramos, A.P., Sidhaye, J., Krishna, A., Solomatina, A., Hoffmann, K.B. et al. (2022) Matrix topology guides collective cell migration *in vivo*. bioRxiv 10.1101/2022.01.31.478442 36208624

[50] Theveneau, E., Steventon, B., Scarpa, E., Garcia, S., Trepat, X., Streit, A. et al. (2013) Chase-and-run between adjacent cell populations promotes directional collective migration. Nat. Cell Biol. 15, 763–772 10.1038/ncb2772 23770678 PMC4910871

[51] Szabó, A., Melchionda, M., Nastasi, G., Woods, M.L., Campo, S., Perris, R. et al. (2016) *In vivo* confinement promotes collective migration of neural crest cells. J. Cell Biol. 213, 543–555 10.1083/jcb.201602083 27241911 PMC4896058

[52] Bajanca, F., Gouignard, N., Colle, C., Parsons, M., Mayor, R. and Theveneau, E. (2019) *In vivo* topology converts competition for cell-matrix adhesion into directional migration. Nat. Commun. 10, 1518 10.1038/s41467-019-09548-5 30944331 PMC6447549

[53] Barriga, E.H., Franze, K., Charras, G. and Mayor, R. (2018) Tissue stiffening coordinates morphogenesis by triggering collective cell migration *in vivo*. Nature 554, 523–527 10.1038/nature25742 29443958 PMC6013044

[54] Shellard, A. and Mayor, R. (2021) Collective durotaxis along a self-generated stiffness gradient *in vivo*. Nature 600, 690–694 10.1038/s41586-021-04210-x 34880503

[55] Wildenberg, G.A., Dohn, M.R., Carnahan, R.H., Davis, M.A., Lobdell, N.A., Settleman, J. et al. (2006) p120-catenin and p190RhoGAP regulate cell-cell adhesion by coordinating antagonism between Rac and Rho. Cell 127, 1027–1039 10.1016/j.cell.2006.09.046 17129786

[56] Hashimoto, H. and Munro, E. (2019) Differential expression of a classic cadherin directs tissue-level contractile asymmetry during neural tube closure. Dev. Cell 51, 158–172.e4 10.1016/j.devcel.2019.10.001 31639367 PMC7458479

[57] Koser, D.E., Thompson, A.J., Foster, S.K., Dwivedy, A., Pillai, E.K., Sheridan, G.K. et al. (2016) Mechanosensing is critical for axon growth in the developing brain. Nat. Neurosci. 19, 1592–1598 10.1038/nn.4394 27643431 PMC5531257

[58] Song, Y., Li, D., Farrelly, O., Miles, L., Li, F., Kim, S.E. et al. (2019) The mechanosensitive ion channel Piezo inhibits axon regeneration. Neuron 102, 373–389.e6 10.1016/j.neuron.2019.01.050 30819546 PMC6487666

[59] Pieuchot, L., Marteau, J., Guignandon, A., Dos Santos, T., Brigaud, I., Chauvy, P.-F. et al. (2018) Curvotaxis directs cell migration through cell-scale curvature landscapes. Nat. Commun. 9, 3995 10.1038/s41467-018-06494-6 30266986 PMC6162274

[60] Luciano, M., Xue, S.-L., De Vos, W.H., Redondo-Morata, L., Surin, M., Lafont, F. et al. (2021) Cell monolayers sense curvature by exploiting active mechanics and nuclear mechanoadaptation. Nat. Phys. 17, 1382–1390 10.1038/s41567-021-01374-1

[61] Reversat, A., Gaertner, F., Merrin, J., Stopp, J., Tasciyan, S., Aguilera, J. et al. (2020) Cellular locomotion using environmental topography. Nature 582, 582–585 10.1038/s41586-020-2283-z 32581372

[62] Vecchio, L., Thiagarajan, S., Caballero, R., Vigon, D., Navoret, V., Voituriez, L. (2020) Collective dynamics of focal adhesions regulate direction of cell motion. Cell Syst. 10, 535–542.e4 10.1016/j.cels.2020.05.005 32553185

[63] Bergert, M., Erzberger, A., Desai, R.A., Aspalter, I.M., Oates, A.C., Charras, G. et al. (2015) Force transmission during adhesion-independent migration. Nat. Cell Biol. 17, 524–529 10.1038/ncb3134 25774834 PMC6485532

[64] Shellard, A., Hampshire, P.A.E., Stillman, N.R., Dix, C., Thorogate, R., Imbert, A. et al. (2023) Frictiotaxis underlies adhesion-independent durotaxis. bioRxiv 10.1101/2023.06.01.543217 PMC12019219 40268931

[65] Ratheesh, A., Biebl, J., Vesela, J., Smutny, M., Papusheva, E., Krens, S.F.G. et al. (2018) Drosophila TNF modulates tissue tension in the embryo to facilitate macrophage invasive migration. Dev. Cell 45, 331–346.e7 10.1016/j.devcel.2018.04.002 29738712

[66] Belyaeva, V., Wachner, S., Gyoergy, A., Emtenani, S., Gridchyn, I., Akhmanova, M. et al. (2022) Fos regulates macrophage infiltration against surrounding tissue resistance by a cortical actin-based mechanism in Drosophila. PLoS Biol. 20, e3001494 10.1371/journal.pbio.3001494 34990456 PMC8735623

[67] Akhmanova, M., Emtenani, S., Krueger, D., Gyoergy, A., Guarda, M., Vlasov, M. et al. (2022) Cell division in tissues enables macrophage infiltration. Science 376, 394–396 10.1126/science.abj0425 35446632

[68] Mishra, A.K., Campanale, J.P., Mondo, J.A. and Montell, D.J. (2019) Cell interactions in collective cell migration. Development 146, dev172056 10.1242/dev.172056 31806626 PMC7375824

[69] Montell, D.J., Yoon, W.H. and Starz-Gaiano, M. (2012) Group choreography: mechanisms orchestrating the collective movement of border cells. Nat. Rev. Mol. Cell Biol. 13, 631–645 10.1038/nrm3433 23000794 PMC4099007

[70] Cai, D., Chen, S.-C., Prasad, M., He, L., Wang, X., Choesmel-Cadamuro, V. et al. (2014) Mechanical feedback through E-cadherin promotes direction sensing during collective cell migration. Cell 157, 1146–1159 10.1016/j.cell.2014.03.045 24855950 PMC4118667

[71] Penfield, L. and Montell, D.J. (2022) Nuclei function as wedges to pry open spaces and promote collective, confined cell migration *in vivo*. bioRxiv 10.1101/2021.12.16.473064

[72] Smutny, M., Ákos, Z., Grigolon, S., Shamipour, S., Ruprecht, V., Čapek, D. et al. (2017) Friction forces position the neural anlage. Nat. Cell Biol. 19, 306–317 10.1038/ncb3492 28346437 PMC5635970

[73] Dalle Nogare, D.E., Natesh, N., Vishwasrao, H.D., Shroff, H. and Chitnis, A.B. (2020) Zebrafish posterior lateral line primordium migration requires interactions between a superficial sheath of motile cells and the skin. eLife 9, e58251 10.7554/eLife.58251 33237853 PMC7688310

[74] Liu, Y.-J., Le Berre, M., Lautenschlaeger, F., Maiuri, P., Callan-Jones, A., Heuzé, M. et al. (2015) Confinement and low adhesion induce fast amoeboid migration of slow mesenchymal cells. Cell 160, 659–672 10.1016/j.cell.2015.01.007 25679760

[75] Huljev, K., Shamipour, S., Pinheiro, D., Preusser, F., Steccari, I., Sommer, C.M. et al. (2023) A hydraulic feedback loop between mesendoderm cell migration and interstitial fluid relocalization promotes embryonic axis formation in zebrafish. Dev. Cell 58, 582–596.e7 10.1016/j.devcel.2023.02.016 36931269

[76] Moreau, H.D., Blanch-Mercader, C., Attia, R., Maurin, M., Alraies, Z., Sanséau, D. et al. (2019) Macropinocytosis overcomes directional bias in dendritic cells due to hydraulic resistance and facilitates space exploration. Dev. Cell 49, 171–188.e5 10.1016/j.devcel.2019.03.024 30982662

[77] Bera, K., Kiepas, A., Godet, I., Li, Y., Mehta, P., Ifemembi, B. et al. (2022) Extracellular fluid viscosity enhances cell migration and cancer dissemination. Nature 611, 365–373 10.1038/s41586-022-05394-6 36323783 PMC9646524

[78] Mitchel, J.A., Das, A., O'Sullivan, M.J., Stancil, I.T., DeCamp, S.J., Koehler, S. et al. (2020) In primary airway epithelial cells, the unjamming transition is distinct from the epithelial-to-mesenchymal transition. Nat. Commun. 11, 5053 10.1038/s41467-020-18841-7 33028821 PMC7542457

[79] Riching, K.M., Cox, B.L., Salick, M.R., Pehlke, C., Riching, A.S., Ponik, S.M. et al. (2014) 3D collagen alignment limits protrusions to enhance breast cancer cell persistence. Biophys. J. 107, 2546–2558 10.1016/j.bpj.2014.10.035 25468334 PMC4255204

